# Targeted Interdisciplinary Model for Evaluation and Treatment of Neuropsychiatric Symptoms (TIME) in home care services: a cluster randomized feasibility trial

**DOI:** 10.1186/s12913-022-07830-9

**Published:** 2022-03-29

**Authors:** Kari-Anne Hoel, Bjørn Lichtwarck, Anette Væringstad, Ingvild Hjorth Feiring, Anne Marie Mork Rokstad, Geir Selbæk, Jūratė Šaltytė Benth, Sverre Bergh

**Affiliations:** 1 The Research Centre for Age-Related Functional Decline and Disease, Innlandet Hospital Trust, Ottestad, Norway; 2 Faculty of Medicine, University of Oslo, Oslo, Norway; 3 The Norwegian National Centre for Ageing and Health, Vestfold Hospital Trust NO, Tønsberg, Norway; 4 Faculty of Health and Social Sciences, Molde University College, Molde, Norway; 5 Department of Geriatric Medicine, Oslo University Hospital, Oslo, Norway; 6 Institute of Clinical Medicine, Campus Ahus, University of Oslo, Oslo, Norway; 7 Health Services Research Unit, Akershus University Hospital, Nordbyhagen, Norway

**Keywords:** Dementia, Nonpharmacological interventions, Psychosocial interventions, Person-centered care, Depression, Anxiety, Neuropsychiatric symptoms, BPSD, Community-dwelling patients, Home care services

## Abstract

**Background:**

Behavioral and psychological symptoms of dementia (BPSD) occur frequently in people with dementia and can contribute to an increased need for help and a reduced quality of life, but also predict early institutionalization. The Targeted Interdisciplinary Model for Evaluation and Treatment of Neuropsychiatric Symptoms (TIME) might be a useful personalized approach to BPSD in people with dementia. The main objective of this feasibility trial was to explore the trial design and methods along with the patients’ and the home care staff’s acceptance of the TIME intervention before developing a definitive trial. Additionally, we wanted to explore whether TIME could be appropriate for staff in home care services in their approach towards people with dementia with anxiety and depression.

**Methods:**

This was a 18-month feasibility trial using a parallel cluster randomized controlled design. Nine municipalities from the eastern part of Norway (clusters) — 40 people with dementia and 37 of their next of kin— were randomized to the TIME intervention or to treatment as usual. In addition, qualitative data as field notes were collected and summarized.

**Results:**

The staff in home care services experienced TIME as an appropriate method; in particular, the systematic approach to the patient’s BPSD was experienced as useful. However, the completion of the assessment phase was considered exhaustive and time-consuming, and some of the staff found it challenging to find time for the case conferences.

**Conclusions:**

We consider that TIME, with some adjustments, could be useful for staff in home care services in cases where they face challenges in providing care and support to people with dementia. This feasibility trial indicates that we can move forward with a future definitive randomized controlled trial (RCT) to test the effect of TIME in people with dementia receiving home care services.

**Trial registration:**

ClinicalTrial.gov identifier: SI0303150608.

## Background

The number of people with dementia is expected to increase rapidly in the coming years, from 47 million worldwide in 2015 to 135 million in 2050 [1]. In line with this, an approximately 130% increase of people with dementia is expected in Norway over the next 30 years [2]. As the disease progresses, people with dementia will need comprehensive care and support, and both the national and international contexts see a desire and a need for people with dementia to receive essential healthcare services at home [3–5]. In Norway, the home care services provide assistance when people living at home need healthcare services. Service should be provided based on an assessment of the patient's need. Health-related care and support is free of charge for the patient, but services are limited by the availability of resources. The schedule of the day and practical tasks might be prioritized over an individually tailored service [6], and this might challenge the services’ ability to manage behavioral and psychological symptoms of dementia (BPSD). These symptoms can contribute to increased need for help, reduced quality of life (QoL), increased suffering for the patient, and early institutionalization [7–9]. In addition, BPSD may be stressful for relatives [8–13] and challenging for health care staff to handle as they provide the necessary care and support [9, 13].

Most people with dementia will experience BPSD during the course of the disease [9, 10, 14, 15]. A person-centered approach with care and support that is tailored to the individual needs of the patients are recommended over pharmacological treatment [15–17]. In general the BPSD symptoms anxiety and depression are symptoms that occur frequently in patients with dementia [10, 15, 18, 19] and reduce the person's QoL, worsen their cognition, and impair their physical function [15]. For people with dementia who receive home care services, BPSD are common. And although the symptoms may be difficult to detect, these symptoms affect the daily lives of people with dementia and are therefore important for home care staff to treat [20, 21]. Effective non-pharmacological interventions for people with BPSD living at home are needed [15, 17], and they should be offered as a first choice to people with dementia who have BPSD. A systematic person-centered approach to BPSD might enable staff to tailor care and support to the individual patient [22]; however few studies have examined interventions used by staff in home care services to manage these symptoms.

### The Target Interdisciplinary Model for Evaluation and Treatment of Neuropsychiatric Symptoms (TIME)

TIME is a multicomponent biopsychosocial intervention for assessing and treating BPSD in dementia and other complex disorders [23]. The model draws on elements from person-centered care [24] and cognitive behavioral therapy (CBT) [25], and it is intended as a tool for the staff to use in situations where care and support tailored to the individual patient is needed. TIME consists of three overlapping phases. The first phase, the Assessment phase, involves a thorough assessment of the patient, using a range of standardized assessment tools combined with an examination of the patient by a physician. This phase forms the basis for second phase in the model, the Reflection phase: during a scheduled guided case conference lasting 60 to 90 min, staff use systematic reflection based on principles from CBT to draw up personalized measures. In the third phase, the Action and evaluation phase, these measures are systematically implemented and evaluated. TIME was developed for use in nursing homes. In addition to being perceived as useful by the staff as a new method for learning in practice and for mastery of complex problems, the model showed an effect to reduce agitation for people with dementia [22, 26].

The main objective of this feasibility trial was to explore the trial design along with the patients’ and the home care staff’s acceptance of the TIME intervention before the development of a definitive RCT. Additionally, we wanted to explore whether TIME could be an appropriate tool for the staff in home care services for their approach toward people with dementia with anxiety and depression. Primary and secondary outcomes has only been tested to assess whether they are perceived as clinically relevant for staff and patients.

## Method/design

This was a 18-month feasibility trial using a parallel cluster randomized controlled design (RCT). Nine municipalities participated in the project, with each municipality being a cluster. A total of 40 people with dementia and 37 of their next of kin participated in the trial, which lasted from October 2019 to April 2021.

The trial was approved by the Regional Committee for Medical and Health Research Ethics in eastern Norway (REK South-East no. 2018/758). Our paper follows the Consolidated Standards of Reporting Trials (CONSORT) guidelines for reporting randomized pilot and feasibility trials [27]; the trial was registered in July 2019 in clinicaltrials.gov (SI0303150608). No changes in methods or design were made after the trial registration.

### Sample/participants

A convenience sample, based on geographical proximity to the research center, 80 municipalities from the eastern part of Norway were invited to participate in the trial. The 80 municipalities were all in geographical proximity to the research center to reduce travel costs and time. Nine municipalities from five different counties, varying in geographical size and number of inhabitants, agreed to participate. Municipalities were invited either through recruitment meetings or through telephone contact with the leaders of home care services. All leaders of home care services in the municipalities received oral and written information about the trial and signed a collaboration agreement. Municipalities that had previously used TIME or other similar models in their patient-related work were excluded from the trial.

Patients and next of kin were included maximum three days before the baseline assessment; the first patient and next of kin were included October 21, 2019. Each municipality was asked to include at least five patients and the next of kin who knew the patient best. Since this is a feasibility trial, a formal sample size calculation is not necessary as the effect of the outcome in this trial was not considered important [27]. Due to the large number of patients receiving home care services, it was impossible to screen everyone for the inclusion criteria; thus, the staff in the home care service selected patients who, based on a clinical assessment, were potentially eligible for inclusion. These patients were then assessed according to the inclusion criteria. The inclusion criteria for patients were: dementia defined as a score of 1 or more on the Clinical Dementia Rating Scale (CDR) [28], a minimum of 15 minutes of home care services per day for the previous 4 weeks, and affective symptoms defined as a score of ≥ 12 on the Neuropsychiatric Inventory – Nursing Home (NPI-NH) affective subsyndrome (sum of the scores on the depression and anxiety items) [29]. The only exclusion criterion was anticipated shorter life expectancy than three months. The inclusion criterion for next of kin was to have status as a relative in the included patient’s medical record. Otherwise, there was no requirement for next of kin to live with the included patient or to be significant involved in the care provision.

Home care staff who knew the patient, informed them about the trial and ensured that informed written consent was given. The home care staff evaluated each participant’s ability to give consent to participate in the trial. In cases where the patient was considered unable to consent, a next of kin who knew the patient well consented on behalf of the patient. The next of kin of the included participants gave their written consent to participate.

### Data collection and randomization

A baseline assessment of the included patients and the next of kin was conducted from one to seven days before randomization, by eight well experienced and trained project nurses. All project nurses received one day of training in assessing patients and next of kin. The baseline and follow-up assessments were conducted as telephone interviews, wherein the home care staff who knew the patient best answered the assessment based on knowledge of the patient and previously collected data. It was not the same staff who included participants and assessed the inclusion criteria as those interviewed for baseline assessments. Because of this, some participants did not achieve the same NPI-NH score at inclusion and at the baseline assessment. The next of kin were also interviewed by telephone by the same project nurses. The project nurses were blinded to group allocation and were not affiliated with the home care services.

Municipalities were first stratified into small and large municipalities and then randomly assigned 1:1 to either the intervention group or the control group within each stratum. This process was performed by a trial-independent biostatistician. The research team then provided the municipalities with the allocation results. The time that elapsed between baseline assessment and intervention initiation varied from 1 to 7 days.

### Control and intervention phases of the trial

#### Joint education and training of the staff in intervention group and in control group

Before the inclusion of patients and next of kin and before randomization, three staff members from each municipality received one-day training on inclusion procedures. The leaders of home care services were encouraged to select these persons based on their suitability and knowledge of the patients. The training consisted of how to use the necessary assessment tools and how to assess consent for participation from the individual patients.

After randomization, the staff in both the intervention group and the control group received two hours of educational sessions on dementia and BPSD, run by trained project nurses, at the location of the service. The sessions were intended to be at a level that would increase knowledge among unskilled workers and act as a refresher for skilled staff. It was desirable that as many of the staff as possible participated in the training, including leaders, GPs, and other collaborating health personnel. Therefore, the same training was given twice so that as many of the staff as possible had the opportunity to participate (for operational reasons). Educational sessions were given over a period of two weeks. The control group then continued to provide care as usual based on measures previously assigned to the individual patients.

#### Education and training of staff in the intervention group— the intervention

The staff in the intervention group received another three hours of training in TIME, based on the manual’s recommendation [23], which included an instructional film illustrating the case conference and a practical exercise in conducting the case conference. The three staff members from each municipality in the intervention group who had participated in recruiting patients also received three hours of extra training in assessment tools and in how to conduct the case conferences. These three staff members had a special responsibility for the implementation of the intervention in the home care services.

The three phases of TIME were then carried out for each of the patients in the intervention group. To collect personal information and register BPSD in the assessment phase, the various assessment tools recommended in the TIME manual were used [23]. In addition, all patients were examined by a GP and the patient's medications were reviewed. The reflection phase drew on the assessments and a case conference was conducted for all patients in the intervention group. A project nurse with experience in supervising TIME assisted the home care services staff at the first case conference, after which the staff conducted case conferences for the rest of the patients without support. In the action and evaluation phase, measures tailored to the individual patients were implemented and evaluated systematically.

Municipalities in the intervention group received two follow-up telephone calls from a project nurse while the intervention took place. The content of these calls was related to the progress of the assessments of the patients and whether a case conference had been scheduled and/or conducted.

An overview of the course of the intervention is presented in Table 1.

**Table 1:** The course of the intervention

	**The intervention group**	**The control group**
One-day training on inclusion procedures, necessary assessment tools and how to assess consent for participation	X	X
Two hours of educational sessions on dementia and BPSD	X	X
Three hours of educational sessions of training in TIME	X	
Three hours training in assessment tools and how to conduct a case conference (three staff members)	X	
Baseline assessment	X	X
Two follow-up telephone calls during the intervention	X	
Conducting the three phases of TIME	X	
Follow-up assessment	X	X

*BPSD* Behavioural and Psychological Symptoms of Dementia, *TIME* Targeted Interdisciplinary Model for Evaluation and Treatment of Neuropsychiatric Symptoms

### Trial measures

#### Exploring the trial design and methods, and acceptance of the intervention

The feasibility of the trial was assessed based on field notes from three project nurses, and consisted of logs, notes and registrations about the inclusion process of participants and the implementation of the three phases of TIME. The assessment of the implementation of TIME included registration of the staff’s and the leaders’ participation in the educational sessions on dementia and BPSD and training in TIME and the leaders’ participation in the case conferences. In addition we assessed the characteristics of the staff with the special responsibility of implementing of the intervention in the home care services, the collaboration with doctors and other health personnel, as well as the home care services' general ability to learn and use a new intervention such as TIME. All stages of the implementation were continually registered and logged by the three project nurses, and notes were taken on the implementation of case conferences regarding all patients in the intervention group.

#### Assessing whether TIME could be an appropriate tool for staff in their approach towards people with dementia with anxiety and depression

Participants were categorized based on the Clinical Dementia Rating scale, a global assessment tool, into no dementia (CDR 0), mild cognitive impairment (CDR0.5), mild- (CDR 1), moderate- (CDR 2) or severe dementia (CDR 3) [28]. The primary outcome measure was the difference between the intervention group and the control group in changes in symptoms of depression, as measured by the Cornell Scale for Depression in Dementia (CSDD) [30, 31] from baseline to follow-up at 6 months. The secondary outcomes were the differences between the intervention group and the control group in changes from baseline to 6 months in neuropsychiatric symptoms measured with the NPI-NH affective subsyndrome (sum of NPI-NH anxiety and depression scores) and total NPI-NH score, caregiver distress (NPI-NH caregiver distress score)[29], QoL as measured by Quality of life in late-stage dementia (QUALID) [32, 33], burden of care for relatives as measured by the Relative Stress Scale (RSS) [34], and rejection of care as measured by Minimum Data Set (MDS) [35]. The final secondary outcome was the difference between the two groups in the frequency of admission to nursing home between baseline and 18 months.

The CSDD is appropriate for assessing the symptoms of depression in people with dementia, with severity scores for each of the 19 items on a scale of 0–2 for a maximum total score of 38; a score above 7 indicates mild to severe depression [30, 31]. The NPI-NH assess the frequency (range: 0–4) and the severity (range: 0–3) of 12 psychological and behavioral symptoms. A single item score is generated by multiplying frequency by severity, giving a range of 0–12 with a higher score indicating a more severe symptom, and a range in total score for the NPI-NH from 0–144. In the NPI-NH caregiver distress scale, each item is scored from 0–5, with a higher score indicating more severe distress [29]. QUALID consists of 11 single items scored from 1–5 (total scores 11–55), with a lower score indicating a better QoL [32, 33]. The RSS scores range from 0–60, with a higher score indicating more severe distress [34]. The minimum Data Set (MDS) scores from 0–3 where higher scores indicate more frequent rejection of care.

### Data analysis

Our report follows the guidelines for the Consolidated Standards of Reporting Trials (CONSORT) for reporting randomized pilot and feasibility trials [27]. Demographic and clinical characteristics of the patients in the intervention and control groups at baseline are presented as means and standard deviations (SDs), or frequencies and percentages, as appropriate. The results of the feasibility data of the intervention in home care services are presented as frequencies and a summary of the researcher’s field notes. The fieldnotes have a qualitative form and are presented according to the main content of the RCT design and methods; i.e. recruitment procedures, clinical relevance of outcomes, participation and performance of educational sessions, leaders participation, collaboration with the GPs and other health personnel, and the patients’ and the home care staff’s acceptance of the TIME intervention.

All variables used to measure primary and secondary outcomes were described as means and SDs within the two groups at baseline and at follow-up. Cluster effect on the municipality level was assessed by intra-class correlation coefficient. Linear mixed models with random effects for patients nested within municipalities were estimated to assess the differences between the groups in changes in primary and secondary outcomes. The models contained a dummy for time variable (baseline vs. follow up), a dummy for group (intervention vs. control), and the interaction between these two. Post hoc analyses were conducted to derive the mean differences between the group in changes. The models were also adjusted for age and gender.

All tests were two-sided, and results with *p*-values below 0.05 were considered statistically significant. The analyses were performed in SPSS v.26 and SAS v.9.4 by a statistician blinded to group affiliation.

## Results

### Patients

**Fig. 1:**
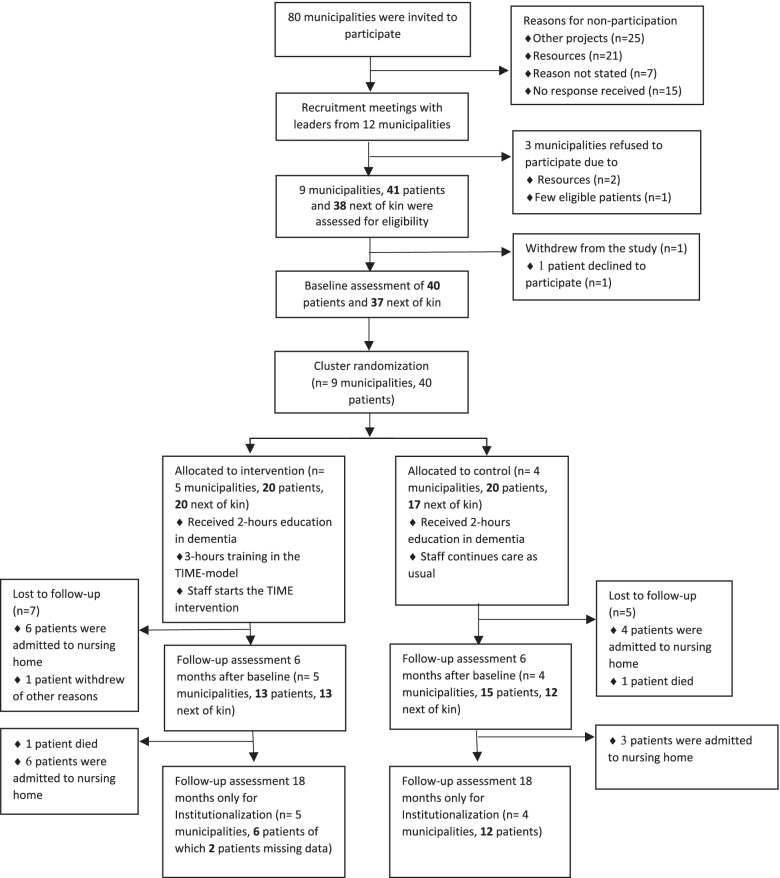
Flowchart of the clusters and individuals throughout the phases of the feasibility trial

According to Fig. 1, nine municipalities agreed to participate in the study. Each municipality was asked to recruit five patients, but some municipalities were not able to include five participants who fulfilled the inclusion criteria. In total, 41 people with dementia and 38 next of kin were included in the trial from 21^st^-23^rd^ October 2019. One participant withdrew the consent after the baseline assessment, leaving 20 patients and 20 next of kin in the intervention group, and 20 patients and 17 next of kin in the control group. Twenty eight participants and 25 next of kin completed the 6 months follow up assessment. Admission to nursing home from baseline to 18 months follow up was 12 patients (30%) in the intervention group and 7 patients (17.5%) in the control group.

The participants were on average 81.7 (SD = 7.9) years old in the intervention group and 81.3 (SD = 6.9) years old in the control group, with a predominance of women in both groups. All participants in the study received home care services daily with 75.0 (SD = 37.0) visits per month in the intervention group and 72.7 (SD = 41.1) visits per month in the control group. Each visit lasted 20.0 (SD = 9.2) minutes in the intervention group and 20.5 (7.1) minutes in the control group (Table 2).

**Table 2:** Description of the home care services and demographic and clinical characteristics of patients at baseline

Variable	Intervention Group (*N* = 20)	Control Group (*N* = 20)
**Demographic and clinical characteristics of patients**		
Age in years, mean (SD)	81.7 (7.9)	81.3 (6.9)
Female, n (%)	15 (75.0)	14 (70.0)
Dementia diagnosis, n (%)	11 (55.0)	11 (55.0)
Clinical Dementia Rating scale, CDR (%)		
No cognitive impairment	0	0
Mild cognitive impairment	0	1 (5.0)
Mild dementia	12 (60.0)	6 (30.0)
Moderate dementia	7 (35.0)	11 (55.0)
Severe dementia	1 (5.0)	2 (10.0)
General Medical Health Rating scale (%)		
Excellent	0	0
Good	9 (45.0)	10 (50.0)
Fair	7 (35.0)	8 (40.0)
Poor	4 (20.0)	2 (10.0)
Cornell Scale for Depression in Dementia (SD)	8.7 (5.5)^1^	9.4 (6.7)^1^
Neuropsychiatric Inventory Nursing Home version (NPI-		
NH) NPI-NH affective subsyndrome score (SD)	10.1 (6.6)	12.6 (7.0)^a^
NPI-NH Sum, (SD)	18.9 (9.6)	29.9 (18.1)
NPI-NH distress scale sum (SD)	6.7 (4.6)	8.4 (6.0)
Quality of Life in Late-stage Dementia Scale, (SD)	22.7 (6.4)	24.6 (7.9)
Relatives Stress Scale, (SD)	22.9 (13.5)	28.3 (12.3)^b^
Rejection of Care MDS-E0800, n (%)		
Behavior not exhibited, (%)	17 (85.0)	10 (50.0)
Behavior of this type occurred 1–3 days, (%)	2 (10.0)	4 (20.0)
Behavior of this type occurred 4–6 days, (%)	1 (5.0)	3 (15.0)
Behavior of this type occurred daily, (%)	0	3 (15.0)
**Description of the home care services**		
Number of visits from home care services last month (SD)	75.0 (37.0)^a^	72.7 (41.1)
Number of hours of help from home care services last month (SD)	27.9 (23.2)^a^	24.5 (14.9)
Number of minutes per visit from home care services (SD)	20.0 (9.2)^a^	20.5 (7.1)
Number of staff in home care services (SD)	73.2 (103.5)^c^	44.3 (23.6)^d^
Number of patients in home care services (SD)	218.0 (109.0)^c^	205.0 (67.7)^d^
Number of staff visiting the patient last week (SD)	10.0 (4.3)^c^	9.5 (4.2)^d^

^a^*N* = 19

^b^*N* = 17

^c^*N* = 5

^d^*N* = 4

### The feasibility of the study

For the joint educational sessions on dementia, 139 staff members from the intervention municipalities and 123 staff members from the control municipalities participated. In the TIME training sessions, 124 staff members from the intervention municipalities participated. Nurses, other health professionals without university education, staff without formal care or health education, and leaders participated in the training. Some municipalities had challenges in scheduling time for the staff to participate—especially for nurses, who had to do patient-related work. Five leaders participated, but no GPs.

Based on the researcher’s field notes, it emerged that most of the staff in the home care services had little or no experience with using the assessment tools before the study started, and the assessment phase was experienced by most as extensive and time-consuming. In general, the staff felt that this phase contributed to better knowledge of the patient and hence a better opportunity to provide tailored care. Each municipality in the intervention group arranged one case conference for each included participant where the staff developed personalized measures for the individual patients. From three to six of the staff attended each of these conferences. It was a consistent experience that finding time for the case conferences was a challenge, which resulted in a delay to conduct the third phase, the action- and evaluation phase, in the model. The last case conference was scheduled close to the follow-up assessment. The patients’ GPs did not attend any of the case conferences. The systematic way of collaborating on the formulation of customized measures in these conferences was perceived by all staff as useful, even though the meetings were perceived as taking too much time for any single patient. In addition, the staff as a whole experienced that if the challenges at stake in the case conferences had to a greater extent affected the daily care of the patient, it would probably have been easier to prioritize a case conference. However, several of the staff assumed that as they became familiar with the model, the duration of the meetings could be reduced.

During the baseline and follow-up assessment, a few unintended consequences arose. A few of the next of kin experienced that the baseline- and follow up interviews were perceived as an additional burden. One next of kin also experienced a difficult situation, wherein suspicious behavior in the person with dementia was provoked due to the next of kin being interviewed.

### Outcome measures for anxiety and depression

Between-group differences in changes from the baseline to the 6-month follow-up for the primary outcome (i.e., symptoms of depression as measured by the CSDD scale) were not significant. The only significant difference was in change in the secondary outcome NPI-NH sum in favor of the control group. The results of the analyses of primary and secondary outcomes are presented in Table 3.

**Table 3:** Descriptive statistics for primary and secondary outcomes. Differences in changes are derived from the linear mixed model adjusting for cluster effect at the level of the municipality (*n* = 28)

Variable	Baseline		Follow-up		Unadjusted differences in change
	Intervention Group (*n* = 20)	Control Group (*n* = 20)	Intervention Group (*n* = 13)	Control Group(*n* = 15)	Mean (95%CL)
*Primary outcome*					
CSDD					
N	19	19	13	15	
Mean (SD)	8.7 (5.5)	9.4 (6.7)	8.5 (6.0)	7.7 (5.3)	-0.80 (-4.61; 3.01)
*Secondary outcomes*					
NPI-NH Subsyndrome affective score					
N	20	19	9	8	
Mean (SD)	10.1 (6.6)	12.6 (7.0)	14.4 (3.7)	12.4 (8.3)	-4.96 (-12.01; 2.08)
NPI-NH sum					
N	20	20	13	15	
Mean (SD)	18.9 (9.6)	29.9 (18.1)	19.3 (14.0)	18.5 (16.2)	-12.55 (-23.86; -1.24)
NPI-NH sum of occupational disruptiveness					
N	20	20	13	15	
Mean (SD)	6.7 (4.6)	8.4 (6.0)	6.0 (7.1)	4.4 (4.6)	-3.47 (-8.44; 1.49)
QUALID					
N	20	20	13	15	
Mean (SD)	22.7 (6.4)	24.6 (7.9)	22.9 (7.9)	23.7 (9.5)	-1.43 (-6.20; 3.33)
RSS					
N	20	17	13	11	
Mean (SD)	22.9 (13.5)	28.3 (12.3)	23.7 (15.6)	23.3 (10.0)	-2.43 (-8.70; 3.84)
	Intervention group (*n* = 20)	Control Group (*n* = 20)	Intervention group (*n* = 13)	Control Group (*n* = 14)	OR (95% CI)
MDS-E0800 Rejection of care, n (%)					
0	17 (85.0)	10 (50.0)	10 (76.9)	9 (64.3)	0.53 (0.07; 4.01)
1/2/3	3 (15.0)	10 (50.0)	3 (23.1)	5 (35.7)	

Negative difference in change means that the change in Control Group is larger than change in Intervention Group

## Discussion

Given that more people with dementia need to live at home and handle BPSD with the necessary care and support, effective non-pharmacological interventions are of great importance [9, 17]. A person-centered approach towards people with dementia are beneficial [15, 36, 37], and the model TIME can be used by staff in tailoring care and support to the individual patient [22]. This cluster randomized feasibility trial was therefore designed with a pragmatic approach [38], to explore the trial design and methods, and whether TIME was perceived as suitable for tailored care and support for people with dementia with anxiety and depression receiving home care services.

### Trial design and methods

One of the objectives of this trial was to test the trial design and methods, before proceeding with a possible definitive RCT. For the recruitment of municipalities, where the pre-defined number in the trial was ten, our research team experienced it challengeing and time-consuming to recruit enough municipalities. It turned out that many municipalities had ongoing projects and few available resources for participation in research trials. The leaders in the home care services also perceived that TIME could be to extensive to implement with their available resourses. This is an important experience and may have consequences for the design of a definitive RCT. Nevertheless, we cannot ignore the need for the implementation of multi-component and to some extent complex interventions in the development of customized services for people with dementia [15]. However, the intervention has to be balanced with the home care services' available resources. In accordance with the limited time in the service, less time should be scheduled for each case conference, as well as a more targeted and simplified assessment phase adapted to the individual patient.

In this feasibility trial we also wanted to explore whether TIME could be an appropriate tool for the approach of the staff in home care services when caring for people with dementia with affective symptoms, anxiety, and depression. Anxiety and depression were chosen as inclusion criteria, and symptoms of depression as primary outcome, as these symptoms frequently occur in people with dementia [10, 15, 18, 19]. These symptoms also affect a person's daily life and reduce their QoL [15]. However, staff found it difficult to identify patients with these symptoms, as patients with these symptoms rarely were perceived as difficult to help. In addition, other symptoms were perceived by staff as more important to treat, like for example rejection of care from the patients and other care challenges. Thus, the home care service staff experienced that they had few patients who fulfilled the inclusion criteria. This is another important finding in this trial which indicates that this inclusion criterion was not perceived as clinically relevant for a definitive RCT trial, but could also indicate that the staff may benefit from further training in identifying these symptoms in people with dementia.

Staff also conveyed that the inclusion criterion of 15 min of daily home care services — which was intended to ensure that the staff knew the patient well — resulted in fewer patients that met the inclusion criteria. This inclusion criterion may have contributed to exclusion of patients with relevant symptoms, due to shorter visits from home care services than 15 min a day. In a future final RCT it should be considered whether the staff themselves can assess the extent of their knowledge of the patient rather than using predetermined allocated time within each patient as an inclusion criterion. Based on these experiences, it might be important that the inclusion criterion in a final RCT trial is more closely linked to what the staff consider relevant.

### The intervention of TIME

TIME was experienced by the staff in home care services as a systematic way to approach BPSD in people with dementia. This trial indicates that systematic examination and assessment of the patient, combined with scheduled case conferences to discuss measures tailored to the individual patient, might contribute to a tailored care and support to the individual patient.

Scheduling time for the educational sessions and the training in TIME was prioritized in all included municipalities, and conducted as planned. Nevertheless, it seemed difficult to enable nurses to participate, due to their work demands in the home care services. Previous research shows the importance of leader's participation in implementation of person-centered care [39]. It is possible that if all leaders had committed themselves to participating in the trial, the implementation in some municipalities would have been more effective and timely. In a final RCT, it may therefore be necessary to clarify in advance the nurses' and leaders’ expected degree of participation both in the educational sessions and the training in TIME which is probably important for a successful implementation.

Since TIME is an interdisciplinary model, the staff in home care services should collaborate with GPs and other health personnel in tailoring care and support to the individual patient [23]. As part of the assessment phase all patients were examined by a GP. But although collaboration between health professionals is recommended for people with dementia [40], there was a consistent experience among the staff that the collaboration, with especially the GPs, was challenging. Despite the fact that all GPs received information about the study from the research team and were invited by the home care services to participate, no GP participated in the case conferences. In a final RCT trial it should be taken into account that the staff in the home care service often work independently and often without the expected collaboration with other health personnel, like the GPs.

It seemed that the primary outcome, difference in changes of symptoms of depressjon, was probably not clinically relevant for neither the patient nor the staff. The mean value of CSDD was low at baseline, approximately 9, and that clinical depression is seen mainly from a score of 8 and above. This indicates that the participants had mild depressive symptoms and the possibility of affecting these symptoms was probably small. In addition, these may be symptoms that the staff to a small extent consider important compared with other challenges in everyday life. It might therefore be more appropriate to use a more individual goaloriented primary outcome to compromise the heterogeneity of the symptoms and challenges in people with dementia (41).

Although the effectiveness of the TIME intervention itself was not the primary goal of this trial, the only significant difference in change in outcomes was in favor of the control group in the total NPI-NH score, a secondary outcome. The participants had only mild symptoms of depression and a change can therefore be challenging to change by a intervention. However, the outcomes measured in this trial were part of the feasibility trial to assess whether they could be appropriate to use in a definitive RCT. Few participants were included in the study, with no power calculation, and several patients were lost to follow-up. Based on these findings, the effectiveness results of this trial have limited evidence value.

Although this study shows that it might be necessary to adjust the TIME model for use in the home services, it may be unfortunate to make major changes based on the sample in this feasibility trial. A definitive trial alongside a process evaluation of the trial, may therefore be necessary to assess whether the TIME model can and should be recommended for use in the home care services, and what adjustments that may be relevant based on the home care service's available resources.

In this trial we experienced few unintended consequences. However, it should be mentioned that some next of kin were put in challenging situations, wherein inclusion in this trial added to an already large care burden. Some patients were suspicious and did not like questions being asked to the next of kin. This reminds us that asking for extra effort from relatives, such as two telephone interviews, can be perceived by some as demanding.

### Limitations and strengths

A strength of the study was the close contact with the municipalities, where project nurses attended the first case conference in each municipality in the intervention group. Telephone calls from the same project nurses during the intervention period, and the staff’s opportunity to get in touch with project nurses if they had any questions, ensured that TIME and the project itself were carried out as intended. In addition, the implementation of the intervention was well documented by the research team using field notes during the entire trial.

The baseline and follow-up assessments were performed by trained project nurses who were blinded to group allocation and were not affiliated with the home care services. Although some uncertainty may arise whether the interviewed staff knew the patient well enough to answer the questions in the assessment tools, the project nurses who carried out the assessments had experience in assessment through telephone interviews and the necessary expertise in each assessment form.

A limitation of the study was the experienced challenge with including enough municipalities and probably only the municipality most interested in good quality of care took part in the study. Implementing TIME in other municipalities could therefore be more difficult. Another limitation of the study is the large dropout during the trial period, which may indicate that the participants’ health conditions were severe and that they needed nursing home admission earlier than expected. In addition, this trial was in its final phase when the covid-19 pandemic occurred, and several of the included municipalites reported that the staff from the home care services were reassigned to crisis management and planning of pandemic measures. Furthermore, many patients received reduced services from the home care services primarly to prevent the spread of infection. This may have affected the services’ ability to implement the tailored measures that were decided upon in the case conferences, thus potentially affecting the feasibility of the intervention.

## Conclusion

Regarding the trial design, more appropriate inclusion criteria and more clinically relevant outcomes should be considered before a definitive RCT. According to the staff, this could be patient symptoms that presented challenges in providing necessary care and support. For a future trial we therefore recommend using a more individual goal oriented primary outcome compromising the heterogeneity of the symptoms and challenges in people with dementia.The systematic way of working, combined with the thorough assessment of the patient and the reflection on adopted measures, were experienced by staff in the home care services as useful. Experiences from this feasibility trial indicate that we can move forward with a future definitive RCT, testing the effectiveness of TIME for people with dementia receiving home care services.

## Data Availability

The datasets used and/or analyzed in the current study are available from the corresponding author on reasonable request.

## Abbreviations

RCT: Randomized Controlled Trial
QoL: Quality of life
BPSD: Behavioral and Psychological Symptoms in Dementia
CDR: Clinical Dementia Rating scale
NPI-NH: Neuropsychiatric Inventory–Nursing Home Version
TIME: Targeted Interdisciplinary Model for Evaluation and Treatment of Neuropsychiatric Symptoms
GP: General Practitioners
CSDD: Cornell Scale for Depression in Dementia
QUALID: Quality of life in Late-Stage Dementia
RSS: Relative Stress Scale
MDS: Minimum Data Set
SDs: Standard Deviations
CBT: Cognitive behavioral therapy
