# Correction: Targeted Interdisciplinary Model for Evaluation and Treatment of Neuropsychiatric Symptoms (TIME) in home care services: a cluster randomized feasibility trial

**DOI:** 10.1186/s12913-022-08308-4

**Published:** 2022-07-14

**Authors:** Kari-Anne Hoel, Bjørn Lichtwarck, Anette Væringstad, Ingvild Hjorth Feiring, Anne Marie Mork Rokstad, Geir Selbæk, Jūratė Šaltytė Benth, Sverre Bergh

**Affiliations:** 1 The Research Centre for Age-Related Functional Decline and Disease, Innlandet Hospital Trust, Ottestad, Norway; 2 Faculty of Medicine, University of Oslo, Oslo, Norway; 3 The Norwegian National Centre for Ageing and Health, Vestfold Hospital Trust NO, Tønsberg, Norway; 4 Faculty of Health and Social Sciences, Molde University College, Molde, Norway; 5 Department of Geriatric Medicine, Oslo University Hospital, Oslo, Norway; 6 Institute of Clinical Medicine, Campus Ahus, University of Oslo, Oslo, Norway; 7 Health Services Research Unit, Akershus University Hospital, Nordbyhagen, Norway

**Correction: BMC Health Serv Res 22,415 (2022)**

**https://doi.org/10.1186/s12913-022-07830-9**

Following publication of the original article [1], the authors identified an error in the affiliations assignment: the first author, Kari-Anne Hoel, should have had the Faculty of Medicine, University of Oslo (affiliation 2), as affiliation in addition to The Research Centre for Age-related Functional Decline and Disease, Innlandet Hospital Trust (affiliation 1).

This error is corrected in the author list of this Correction article and the original article [1] has been updated.
